# The re‐occurrence of cardiomyopathy in propionic acidemia after liver transplantation

**DOI:** 10.1002/jmd2.12119

**Published:** 2020-04-08

**Authors:** Gerard T. Berry, Elizabeth D. Blume, Ann Wessel, Tajinder Singh, Leah Hecht, Deborah Marsden, Inderneel Sahai, Scott Elisofon, Michael Ferguson, Heung Bae Kim, David J. Harris, Didem Demirbas, Mohammed Almuqbil, William L. Nyhan

**Affiliations:** ^1^ Division of Genetics and Genomics, The Manton Center for Orphan Disease Research, Boston Children's Hospital Harvard Medical School Boston Massachusetts USA; ^2^ Department of Cardiology, Boston Children's Hospital Harvard Medical School Boston Massachusetts USA; ^3^ Pediatrics‐Genetics Department, Massachusetts General Hospital Harvard Medical School Boston Massachusetts USA; ^4^ Division of Gastroenterology, Hepatology and Nutrition, Boston Children's Hospital Harvard Medical School Boston Massachusetts USA; ^5^ Division of Nephrology, Boston Children's Hospital Harvard Medical School Boston Massachusetts USA; ^6^ Department of Surgery, Boston Children's Hospital Harvard Medical School Boston Massachusetts USA; ^7^ Department of Pediatrics University of California San Diego La Jolla California USA

**Keywords:** cardiomyopathy, heart failure, heart transplantation, liver transplantation, organic acid disorder, propionic acidemia

## Abstract

Cardiomyopathy is a frequent complication of propionic acidemia (PA). It is often fatal, and its occurrence is largely independent of classic metabolic treatment modalities. Liver transplantation (LT) is a treatment option for severe PA as the liver plays a vital role in metabolism of the precursors that accumulate in patients with PA. LT in PA is now considered to be a long‐lasting and valid treatment to prevent cardiac disease. The subject of this report had severe cardiomyopathy that largely disappeared prior to undergoing a LT. Three years following the transplant, there was recurrence of cardiomyopathy following a surgery that was complicated with a postoperative aspiration pneumonia. On his last hospital admission, he was presented with pulmonary edema and heart failure. He continued with episodes of intractable hypotension, despite maximum inotropic and diuretic support. He died following redirection of care. We conclude that lethal cardiomyopathy may develop several years after successful LT in patients with PA.

**SYNOPSIS**

We report a male with infantile onset propionic acidemia who underwent liver transplantation at 11 years of age and died at 19 years of age due to cardiomyopathy. We conclude that patients with propionic acidemia may develop lethal cardiomyopathy several years after successful liver transplantation.

## 1 INTRODUCTION

Propionic acidemia (PA; OMIM: 606054) is an autosomal recessive inborn error of metabolism due to a deficiency of propionyl‐CoA carboxylase (EC: 6.4.1.3), which catalyzes the conversion of propionyl‐CoA to d‐methylmalonyl‐CoA in the mitochondrial matrix.^1^ This multimeric, biotin‐dependent enzyme has six alpha‐ and six beta‐subunits that are encoded by the *PCCA* (OMIM: 232000) and *PCCB* (OMIM: 232050) genes. The frequency of PA is approximately 1 in 60 000, and the majority of patients are secondary to a *PCCB* gene mutation.^2^

As a consequence of the enzyme deficiency, there is an accumulation of propionyl‐CoA metabolites in blood and urine, such as propionylcarnitine. Urine organic acid analysis reveals elevated levels of 2‐methylcitrate, 3‐hydroxypropionate, tiglylglycine, and propionylglycine as well as lactate in some phenotypes.^1^, ^2^ In the neonatal period, PA manifests as lethargy, poor feeding, vomiting, and hypotonia; if not treated there may be progressive encephalopathy accompanied, usually, by metabolic acidosis with an increased anion gap due to lactic acidosis and ketosis, as well as hyperammonemia and hypoglycemia. A late onset form can present as a metabolic crisis under catabolic stress or with developmental delay, failure to thrive, protein intolerance, hypotonia, movement disorders, intellectual disability, and cardiomyopathy.^2^ Rarely, patients may present with a phenotype in infancy that is largely neurologic in nature due to chronic basal ganglia destruction. This more uncommon phenotype supports the hypothesis that the mechanism of disease in PA is related to secondary mitochondrial dysfunction.

The diagnosis of PA usually takes place within the first few weeks of life. Signs are similar to metabolic intoxication.^3^ Complications of PA include neurological sequelae, pancreatitis, and cardiomyopathy. Intellectual disability has been observed in at least 61% of patients by 3 years of age.^4^ Dilated cardiomyopathy was reported to occur at a mean age of 7 years in 6 out of 26 patients or 23% of patients.^5^

Long‐term management of PA includes reducing accumulation of toxic metabolites by limiting dietary intake of natural protein and supplementation with a medical formula limited in the propiogenic amino acids (isoleucine, valine, threonine, methionine), maintenance of optimal calorie intake, carnitine supplementation, and metronidazole for reduction of propionic acid production by gut bacteria.^1^ By its very nature, it is a progressive disease with no curative therapy. Liver transplantation (LT) results in clinical improvement in patients with PA and largely eliminates acute ketolacticacidotic crises.^6^, ^7^, ^8^, ^9^ However, propionate metabolites still remain elevated.^8^, ^10^ Mortality rates have been noted to decrease from 41%‐90% to 7%‐12% over the past four decades based on relatively small cohort studies.^2^ The mortality rate following LT in patients with PA has been reported around 30%—a figure considered very high. Some liver transplant programs have had no deaths in a 10‐year span.^11^ Cardiomyopathy was noted to resolve in a subset of patients with cardiac disease^5^, ^7^, ^12^, ^13^, ^14^, ^15^ and led to enthusiasm that perhaps LT would also eliminate the development or further progression of cardiomyopathy (Table 1). We describe a patient with PA with an initial atypical neurometabolic phenotype in early infancy, whose cardiomyopathy had virtually disappeared prior to the time of LT, and returned 3 years later leading to his death.

**TABLE 1:** Reversal of cardiomyopathy (CM) in propionic acidemia after liver transplantation (LT)

	Gender	Age at diagnosis of PA	Age at the onset of CM	Age at LT	Age at follow‐up	Reference
1	F	46 days	2 years	2 years 2 months	2 year 9 months	^15^
2	F	10 months	6 years	6.7 years	7 years	^5^
					14 years	^14^
3	F	3 days	9 years	9 years	22 years	^5^
4	M	8 months	16 years	16 years	17 years (70 days post transplantation)	^12^
5	M	26 months	18 years	22 years	33 years	^13^
6	F	N/A	N/A	8.3 years	9.3 years	^14^
7	M	7 days	N/A	8.7 years	10.8 years	^7^

*Note*: N/A: data not available.

## 2 CASE REPORT

A male infant was delivered at term by C‐section due to fetal distress to a 39‐year‐old mother. Weight was 3.714 kg. The APGAR scores were 8 and 9. He was fed with standard infant formula and breast milk. For his first 7 days of life, he remained in the neonatal intensive care unit because of tachypnea and a low heart rate. He was noted to have acidosis, and an elevated level of plasma creatinine. He was fed a soy‐based formula because of frequent emesis and a persistent rash in the first month of life.

At 4 months, abnormal movements of the head and eye were observed. They were thought to be seizures and he was eventually started on anti‐epileptic medication. It is possible that this was solely an involuntary movement disorder. Plasma ammonia was elevated. At 5 months of age, urine organic acid analysis demonstrated elevated propionylglycine (38.3 mmol/mol creatinine). Plasma propionic acid (15.0 μmol/L; normal range: 0.6‐2.4) was also elevated. There were bilateral lesions in the basal ganglia and ultimately, he was confirmed to have developed a severe movement disorder with dystonia and choreoathetosis. The diagnosis of PA was confirmed with finding of low propionyl‐CoA carboxylase activity in cultured skin fibroblasts. Subsequently, he was prescribed a low protein diet supplemented with amino acid mixture devoid of valine, isoleucine, methionine, and threonine. He also received supplements of biotin, riboflavin, thiamine, and carnitine. Because of feeding difficulties, a G‐tube was placed at 6‐months of age. The patient was enrolled in a dextromethorphan research study (BCH), where he was given 2 mg/kg per day. Urine organic acid analyses showed elevated levels of 3‐hydroxypropionic acid, 2‐methyl‐3‐hydroxybutyric acid, propionylglycine, and tiglylglycine.

At 1 year of age, the Bayley scales of infant development was performed, and the patient scored less than 50 on mental and motor scales. The movement disorder worsened during late infancy. He subsequently entered a growth hormone trial at University of California San Diego. Analysis of plasma acylcarnitines showed an elevated level of propionylcarnitine (78.1 μmol/L; reference range: <1.78). A murmur was first heard at 2 6/12 years of age at a routine clinic visit (Table S1). Analysis of plasma amino acids showed glycine level of 1602 μmol/L (reference range: 133‐389). Chronic oral therapy with sodium benzoate was started at the age of 7 years. The objective was to lower the glycine concentration to normal. None of the treatment modalities had any major impact on the chronic basal ganglia deterioration, and by 9 years of age he manifested hypotonia, choreoathetoid movements, and dystonia. He also had brisk deep tendon reflexes. Because of hyperammonemia, he was also placed on sodium benzoate. Other medications that were used during childhood included: ubiquinol (400 mg/day), thiamine (25 mg/day), vitamin C (500 mg /day), biotin (10 mg/day), levocarnitine (1000 mg/day), Vitamin E, leucovorin, and cyanocobalamin.

The patient's first episode of overt cardiomyopathy was when he was at an outside hospital and received a baclofen pump. He suffered a *Staphylococcus* parameningeal infection after this procedure, which was associated with the first acute episode of cardiomyopathy when he was 9 8/12 years old (Table S1). The pump was eventually removed. He was judged to not be a good candidate for heart transplant, but eventually achieved stability with medical management. Following aggressive therapy with inotropes and diuretics, the cardiomyopathy largely disappeared. Several months later he had an echocardiogram showing normalization of left ventricular function with ejection fraction of 65% and shortening fraction of 30%. He was then listed for liver transplant and this was then performed at 10 1/12 years of age (Table S1). A pretransplant brain MRI revealed bilateral striatal lesions (Figure S1). He was able to tolerate the liver transplant extremely well from a cardiac standpoint. His ejection fraction was 59% during the transplant admission. He did require a steroid pulse for 3 days for abnormal liver enzymes, and concern for acute cellular rejection. In addition, he developed significant hypertension. He had a seizure, along with hypertension on post‐operative day 16. MRI was suggestive of posterior reversible encephalopathy syndrome. In addition to the immunosuppressive medications, he remained on the same medications. Despite these immediate complications, he was able to be discharged on postoperative day 23. One additional episode of mild, early acute cellular rejection brought about the addition of mycophenolate mofetil to the immunosuppression regimen. There were no further episodes of acute metabolic decompensation and ketolacticacidosis.

The patient had slightly abnormal plasma creatinine level prior to transplantation, but this worsened post‐transplant. He developed chronic kidney disease that was thought to be secondary to the immunosuppressive therapy and left ventricular dysfunction. At 13 6/12 years of age, he underwent deep brain stimulation surgery for his extra‐pyramidal movement disorder. Postoperatively, he suffered his second acute episode of CM following an aspiration event (Table S1). Because of heart failure, there was a major escalation in the number of medications that he was given. Cardiac function improved but required management with chronic oral heart failure therapy. His kidney function failed to normalize.

At 14 11/12 years of age, he presented with cardiogenic shock and pulmonary edema. The echocardiogram showed a left ventricular ejection fraction of 30%. Analyses of plasma acylcarnitine concentrations revealed a marked elevation of propionylcarnitine (C3) to 60 μmol/L (normal: 0‐0.87). The level of 2‐Methylcitrate in serum was markedly elevated at 56 386 nmol/L (normal range: 60‐228). It had been twice that prior to LT. Glycine concentration was 1726 μmol/L (normal: 133‐389); alanine 876 μmol/L (normal: 216‐497). Urinalysis showed large increases in 3‐OH‐propionic acid, tiglylglycine and methylcitric acid, plus small increases in propionylglycine.

On his final admission at 18 10/12 years of age, he presented with pulmonary edema secondary to heart failure (Table S1). He experienced multiple episodes of intractable hypotension on maximum dosage of inotropes and diuretics. After redirection of care, he died at 19 years of age.

## 3 DISCUSSION

The patient suffered a relapse of cardiomyopathy 3 years after LT. It is well known that LT does not completely eliminate the biochemical defect in PA. We have now learned that a recurrence of a cardiac disease is possible even after a successful LT. This is not the first time that lethal cardiac disease developed or was present after LT in patients with PA (Table 2).^14^ There have been several deaths in the immediate posttransplant period due to the progression of cardiomyopathy or rarely its immediate new development. But this is the first report of cardiomyopathy occurring several years after LT.

**TABLE 2:** (Lethal) cardiomyopathy (CM) in propionic acidemia in the peri‐liver transplantation period

	Gender	Age at LT	Onset of CM post‐LT	Death (days after LT)	Reference
1	N/A	26 months (×2)	N/A	D3	Murphy et al (1992)^a^
2	F	2.2 years (×2)	Day 5	D7	^14^
3	M	3.9 years	Day 14	D30	^14^
4	M	6.5 years	Day 3	D12	^14^

*Note*: N/A: data not available. (×2) indicates that the transplantation performed twice.

Abbreviation: LT: liver transplantation.

a Reported only in abstract form in Leuven, SSIEM, 1992.

Our patient with infantile‐onset PA was transplanted at 11 1/12 years of age, following a period when his cardiomyopathy was in a quiescent phase, in an attempt to permanently eliminate its recurrence. Following the transplant, the patient enjoyed 2 5/12 years without overt heart disease, only to succumb eventually to its relentless progression at 19 years of age. In his lifetime, the appearance or re‐appearance of heart disease almost always seemed to be proceeded by a serious stressor, often infectious, such as a perioperative abscess following a baclopen pump implementation or deep brain stimulation surgery with post‐operative complications. The accumulation of propionyl‐CoA in mitochondria may be necessary but not sufficient for the development of cardiomyopathy. In this secondary mitochondriopathy, other factors that promote its development may be dependent on stress such as the immunological system perturbing cardiomyocyte gene expression and promoting cardiac remodeling. We do not believe that this is a reason to stop or curtail LT in PA. The quality of life for the family improves with LT^2^ as it largely eliminates the recurrent episodes of ketolacticacidosis.^16^ Cardiac transplantation is an option for patients with PA. However, the stress of a heart transplant and its follow‐up would likely result in a major episode of metabolic decompensation unless the recipient already had had a liver transplant. One might consider cardiac transplantation in a patient who has had a liver transplant and is well enough to withstand the rigors of this surgery and follow‐up. This area requires further investigation.

Complications associated with PA may present despite strict compliance with low‐protein diet. Aggressive management is required by patients in the course of acute metabolic decompensations, which may present frequently in the first few years of life.^5^ Thus, frequent hospital visits, and the intensity of treatment may impact the lives of patients and their families. It is believed that younger patients, if managed well, may have a better prognosis resulting from decreased duration of metabolic intoxication and better state of nutrition. LT in patients with PA should be performed at an early stage of the disorder for better outcome.^17^ Renal dysfunction in our patient was present after LT and significantly worsened following the recurrence of cardiomyopathy post‐deep brain stimulation surgery. We hypothesize that hypotension and poor perfusion, along with the deleterious effects of the immunosuppressive agent, played a major role in its pathogenesis. However, it is possible that PA *per se* as a renal cell autonomous toxicity may have also been responsible.

Kasahara has recommended early LT as this has the potential for improving the quality of life.^8^ Three patients transplanted in the Kasahara study had experienced a stabilization in their metabolic status with no episodes of hospitalization due to metabolic decompensation, although there was no marked decrease in the levels of serum propionylcarnitine and urine methylcitrate. These patients did not experience cardiac insufficiency suggesting that transplantation is best applied in PA when the condition is still at its early stage.

Benefits of LT include decrease in the frequency of metabolic decompensation, improved quality‐adjusted life years, increased life expectancy, life‐time cost savings,^9^, ^18^ and reversal of dilated cardiomyopathy.^5^, ^10^ Continuous hemofiltration extracorporeal membrane oxygenation,^8^, ^15^ and left ventricular assist devices have been used while waiting for transplantation.^12^ Quintero et al also described a patient who developed a life‐threatening arrhythmia during an aborted LT, was subsequently transplanted and 18 months later has had no evidence of cardiomyopathy.^19^ Rammohan et al reported a patient with cardiomyopathy and LT but the outcome was unclear.^20^ LT in patients with PA is not curative. It does not completely protect against a metabolic stroke. Life‐long posttransplant management is needed.^8^, ^9^, ^10^ Continued protein restriction and l‐carnitine supplementation after LT is advocated.^8^, ^10^, ^21^

Most episodes of cardiomyopathy are not triggered by *bona fide* metabolic crises with ketolacticacidosis. Early discovery via routine echocardiography is key to effective treatment. Making firm conclusions about LT as an effective treatment for PA is complicated by the low incidence of PA and the limited global clinical experience with in these patients.^22^, ^23^ LT may be an effective treatment for PA but this may not always be the case when cardiomyopathy is involved.

The question of LT as an effective treatment for patients with early onset of severe PA is not resolved.^23^ The benefits of LT are elimination of life threatening metabolic decompensations associated with ketolactacidosis, but the biochemical perturbations are not eliminated. The incidence of hyperammonemia is reduced drastically if not completely. LT may prevent or limit the neural and cognitive complications that are characteristic of the disease.^6^ Of course, there is a risk of mortality and morbidity from LT, which may arise from either the surgery itself, or from the suppressed state of immune system which is required for the survival of the graft. Initially, it was believed that neonates may be at high risk of complications from LT. However, studies within the last decade have shown that neonates and infants record more positive outcomes from LT.^24^ Perhaps all patients with infantile onset PA should undergo LT or a comparable treatment strategy utilizing nucleic acids.

In summary, we report the first case of a patient with infantile onset PA who developed lethal cardiomyopathy 3 years after a liver transplant. Following his transplant, he was stable and it was thought that his cardiomyopathy would never return. The pathogenesis of cardiomyopathy in PA ought to be a research priority.

## Supporting information


**Figure S1**. Brain MRI at 11 years of age pre‐liver transplant showing bilateral T2 hyperintensities in the striatum.

**Table S1**. Major medical and medication events.

## References

[1] Schiff M , de Baulny HO , Dionisi‐Vici C . Branched‐chain organic acidurias/acidaemias In: Saudubray J‐M, Baumgartner M, Walter JH, eds. Inborn metabolic diseases – diagnosis and treatment. 6th ed. Berlin Heidelberg: Springer‐Verlag Berlin Heidelberg; 2016:277‐279.

[2] Shchelochkov OA , Carrillo N , Venditti C . Propionic acidemia In: Adam MP, Ardinger HH, Pagon RA, eds. GeneReviews® [Internet]. Seattle, WA: University of Washington; 2016. 22593918

[3] Wongkittichote P , Ah Mew N , Chapman KA . Propionyl‐CoA carboxylase – a review. Mol Genet Metab. 2017;122:145‐152. 29033250 10.1016/j.ymgme.2017.10.002 PMC5725275

[4] Nizon M , Ottolenghi C , Valayannopoulos V , et al. Long‐term neurological outcome of a cohort of 80 patients with classical organic acidurias. Orphanet J Rare Dis. 2013;8:148. 24059531 10.1186/1750-1172-8-148 PMC4016503

[5] Romano S , Valayannopoulos V , Touati G , et al. Cardiomyopathies in propionic aciduria are reversible after liver transplantation. J Pediatr. 2010;156:128‐134. 19818452 10.1016/j.jpeds.2009.07.002

[6] Barshes NR , Vanatta JM , Patel AJ , et al. Evaluation and management of patients with propionic acidemia undergoing liver transplantation: a comprehensive review. Pediatr Transplant. 2006;10:773‐781. 17032422 10.1111/j.1399-3046.2006.00569.x

[7] Critelli K , McKiernan P , Vockley J , et al. Liver transplantation for propionic acidemia and methylmalonic acidemia: perioperative management and clinical outcomes. Liver Transpl. 2018;24:1260‐1270. 30080956 10.1002/lt.25304

[8] Kasahara M , Sakamoto S , Kanazawa H , et al. Living‐donor liver transplantation for propionic acidemia. Pediatr Transplant. 2012;16:230‐234. 22151065 10.1111/j.1399-3046.2011.01607.x

[9] Vara R , Turner C , Mundy H , et al. Liver transplantation for propionic acidemia in children. Liver Transplant. 2011;17:661‐667. 10.1002/lt.22279 21618686

[10] Yorifuji T , Kawai M , Mamada M , et al. Living‐donor liver transplantation for propionic acidaemia. J Inherit Metab Dis. 2004;27:205‐210. 15159651 10.1023/B:BOLI.0000028778.54210.13

[11] Lacaille F . Liver transplantation and liver cell transplantation. Clin Res Hepatol Gastroenterol. 2012;36:304‐307. 22541063 10.1016/j.clinre.2012.03.029

[12] Ameloot K , Vlasselaers D , Dupont M , et al. Left ventricular assist device as bridge to liver transplantation in a patient with propionic acidemia and cardiogenic shock. J Pediatr. 2011;158:866‐867. 21324476 10.1016/j.jpeds.2010.12.031

[13] Arrizza C , De Gottardi A , Foglia E , et al. Reversal of cardiomyopathy in propionic acidemia after liver transplantation: a 10‐year follow‐up. Transpl Int. 2015;28:1447‐1450. 26358860 10.1111/tri.12677

[14] Charbit‐Henrion F , Lacaille F , McKiernan P , et al. Early and late complications after liver transplantation for propionic acidemia in children: a two centers study. Am J Transplant. 2015;15:786‐791. 25683683 10.1111/ajt.13027

[15] Sato S , Kasahara M , Fukuda A , et al. Liver transplantation in a patient with propionic acidemia requiring extra corporeal membrane oxygenation during severe metabolic decompensation. Pediatr Transplant. 2009;13:790‐793. 19207227 10.1111/j.1399-3046.2008.01029.x

[16] Chapman KA , Summar ML , Enns GM . Propionic acidemia: to liver transplant or not to liver transplant? Pediatr Transplant. 2012;16:209‐210. 22332632 10.1111/j.1399-3046.2012.01649.x

[17] Kim TW , Hall SR . Liver transplantation for propionic acidaemia in a 14‐month‐old male. Paediatr Anaesth. 2003;13:554‐556. 12846721 10.1046/j.1460-9592.2003.01104.x

[18] Li M , Dick A , Montenovo M , et al. Cost‐effectiveness of liver transplantation in methylmalonic and propionic acidemias. Liver Transplant. 2015;21:1208‐1218. 10.1002/lt.24173 25990417

[19] Quintero J , Molera C , Juamperez J , et al. The role of liver transplantation in propionic acidemia. Liver Transpl. 2018;24:1736‐1745. 30242960 10.1002/lt.25344

[20] Rammohan A , Gunasekaran V , Reddy MS , Rela M . The role of liver transplantation in propionic acidemia. Liver Transpl. 2019;25:176‐177. 30375139 10.1002/lt.25373

[21] Saudubray JM , Touati G , Delonlay P , et al. Liver transplantation in propionic acidaemia. Eur J Pediatr. 1999;158(suppl 2):S65‐S69. 10603102 10.1007/pl00014325

[22] Leonard JV , Walter JH , McKiernan PJ . The management of organic acidaemias: the role of transplantation. J Inherit Metab Dis. 2001;24:309‐311. 11405351 10.1023/a:1010395724012

[23] McKiernan P . Liver transplantation and cell therapies for inborn errors of metabolism. J Inherit Metab Dis. 2013;36:675‐680. 23296369 10.1007/s10545-012-9581-z

[24] Grabhorn E , Ganschow R , Helmke K , et al. Liver transplantation in infants younger than 6 months old. Transplant Proc. 2002;34:1964‐1965. 12176647 10.1016/s0041-1345(02)03141-x

