# Supplementary material for: The re‐occurrence of cardiomyopathy in propionic acidemia after liver transplantation
Source: JIMD Rep. 2020 Apr 8;54(1):3–8. doi: 10.1002/jmd2.12119 (PMC7358669; doi:10.1002/jmd2.12119)
Supplement: Supplementary file 1 — Figure S1. Brain MRI at 11 years of age pre‐liver transplant showing bilateral T2 hyperintensities in the striatum. [file JMD2-54-3-s001.pptx]

## Slide 1
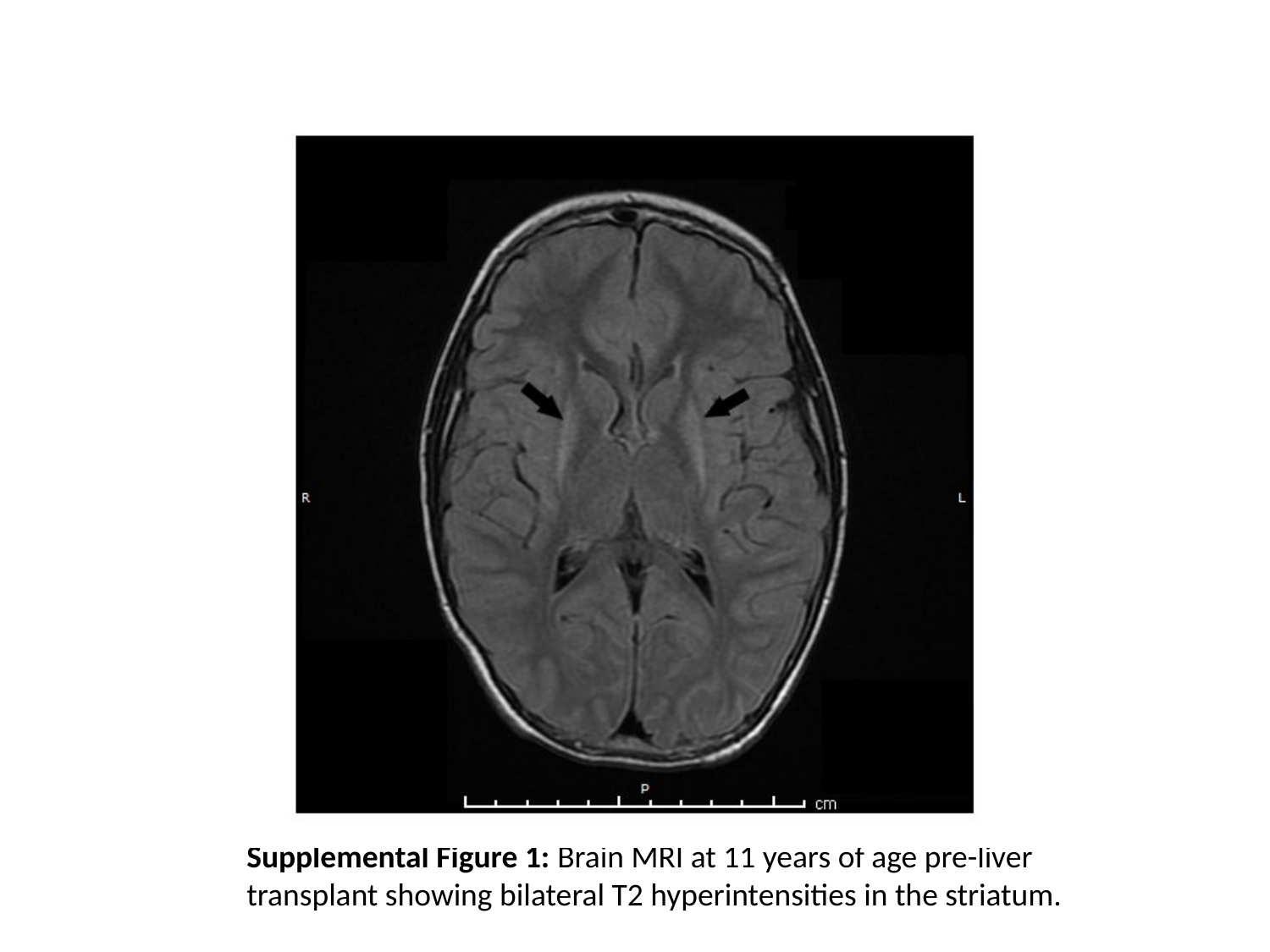

Supplemental Figure 1: Brain MRI at 11 years of age pre-liver transplant showing bilateral T2 hyperintensities in the striatum.
