# Supplementary material for: The re‐occurrence of cardiomyopathy in propionic acidemia after liver transplantation
Source: JIMD Rep. 2020 Apr 8;54(1):3–8. doi: 10.1002/jmd2.12119 (PMC7358669; doi:10.1002/jmd2.12119)
Supplement: Supplementary file 2 — Table S1. Major medical and medication events. [file JMD2-54-3-s002.docx]

***Supplemental table 1: Major Medical and Medication Events***

| ***Age*** | ***Medical event*** | ***List of Medications*** |
| --- | --- | --- |
| 2 and 6/12 years | **1st evidence of heart murmur**  Clinic visit- well  Intermittent emesis  Poor oral intake (gags on food)  G-tube  Constipation  Global developmental delay  Pulls himself to stand  Walks around furniture  No independent steps  Low protein diet  Amino acid mixture  Overnight continuous infusion of a formula with carbohydrates and fats  Growth hormone clinical trial (UCSD)  Dextromethorphan clinical trial (BCH)  Weight-15.7 kg (90th percentile)  Height-94.9 cm (50th-75th percentile)  Head circumference - 49.25 cm (25th percentile)  Grade 2/6 systolic ejection murmur @left sternal border  Chorea and athetosis  Hypotonia  Trace to small amounts of urinary ketones at home  *Lab results:*  Hemoglobin: 11.9 (11.0-13.3)  Hematocrit: 33.2 (31.5-38.0)  WBC: 9.7 (5.67-9.88)  Plasma ammonia: 41 µmol/L (5-32)  Hemoglobin A1C: 5.4  IGF-1: 93 ng/mL (15-189 ng/mL)  IGFBP-3: 2.62 mg/ L (1.4-3.0)  Plasma prealbumin: 23.7 mg% (14-30)  Plasma T4: 9.3 μg/dl (6-14.2)  Plasma TSH: 3.01 mcu/ml (0.3-5.0)  Plasma total carnitine: 69.7  Plasma glycine: 1284 µmol/L  Plasma leucine: 73 µmol/L  Plasma isoleucine: 31 µmol/L  Plasma valine: 83 µmol/L | - Periactin - L-carnitine - Dextromethorpan - Growth hormone - Nutropin AQ cartridge (10 mg/2 mL; patient gets 1.7 mg subcutaneous, once a day before bedtime QHS |
| 9 and 8/12 years | **Acute CM episode #1**  Intrathecal baclofen pump  *Staphylococcus parameningeal* infection  IV antibiotics  Hypotension  Signs of congestive heart failure  Subsequent echocardiogram --> dilated cardiomyopathy  *Left ventricular ejection fraction of 20% and prolonged corrected QT interval of 480 msec* (10 1/12 years)  *Left ventricular ejection fraction of 26%* (10 1/12 years)  Plasma CK: Normal (10 5/12 years)  Plasma troponin: Normal (10 5/12 years) | *Medications added to treatment plan:*   - Lisinopril (5 mg once daily) - Aldactone |
| 11 and 1/12 years | **Liver transplant**  *Pre-operative information:*  Low protein diet and amino acid mixture  Extrapyramidal movement disorder: chorea, athetosis and dystonia  On admission – well  *Low normal/mildly reduced function with ejection fraction of 65%* (10 5/12 y.o.)  *Low normal/mildly reduced function with ejection fraction of 53%* (10 5/12 y.o.)  *Liver Transplant:*  Cadaveric LT was performed. He developed acute rejection immediately post transplantation that was controlled by increased immunosuppression.  *Post-operative information:*  Post-operative complications of organ rejection/medication-induced hypertension and PRES  Nonconvulsive status epilepticus  Subsequent chronic renal dysfunction due to renal injury/medications  Low protein diet and amino acid mixture  Carbohydrate mixture  Continuous movement disorder while awake  No speech, occasionally makes sounds  Weight: 35.1 kg (30th percentile)  Height: 141.5 cm (31st percentile)  Head circumference: 52.5 cm | *Pre-operative medications:*   - Carvedilol (3.8 mL twice a day) - Lanoxin (125 microg per day) - Baclofen (110 mg twice a day) - Leucovorin (5 mg, 3x a day) - Growth hormone - Nutropin AQ cartridge (10 mg/2 mL; patient gets 1.7 mg subcutaneous, once a day before bedtime QHS) - Lisinopril (5 mg once daily) - Spirinolactone (25 mg once a day) - Coenzyme Q10 (300 mg, 3x a day) - Fluoxetine (5 mg, once per day) - Melatonin (5 mL of a 2.5mg/10 mL solution, before bedtime) - Thiamine hydrochoride (15 mg, once a day) - Carnitine (5 mL, 3x a day) - Fructose (4g, 3x a day) - Vitamin E (400 Units per day) - Riboflavin (25 mg twice a day) - Biotin (10 mg, twice a day) - Zofran (4 mg per tablet, one tablet 3x a day as needed) - MiraLAX - MiraLAX100 (17 g, before bedtime as needed) - Clarithin (5 mg per 5 mL solution, 7 mg per day) - Metronidazole (200 mg a day) - Dextramethorpan (300 mg per day) - Robinul (1 mg twice a day)   *Post-operative medications*  *(1.5 months post op):*   - Tacrolimus (4.1 mg per day) - Prednisolone (12 mg per day) - Carnitine (600 mg , 3x a day) - CMV immuglobulin (x2 weeks) |
| 13 and 6/12 years | **Acute CM episode #2**  *Data prior to surgery:*  MRI Brain without Contrast :  Increased T2 signal within the bilateral putamen, which appear small in size. The brainstem appears slightly thinned and the cerebellar vermis also appear slightly small in size. There is a nonspecific focus of T2 hyperintensity within the right cerebellar hemisphere without evidence of associated tissue loss.  *Deep brain stimulation surgery :*  Post-operative complication- Impaired cardiac contractility  Echocardiogram revealed an ejection fraction of 30% in the setting of flash pulmonary edema (possible aspiraton pneumonitis). Patient begun on Milrinone and Lasix and required some pressor support with dopamine for 3 days to maintain adequate mean arterial pressures.  Acute respiratory failure requiring endotracheal intubation.  He had been intubated for 5 days.  Chest x-ray revealed opacification of the left lung.  He did develop 2 tonic-clonic seizures and received Ativan.  He developed watery diarrhea.  Echocardiogram revealed EF 28% (13 6/12 years)  A repeat echocardiogram 4 days later: EF 52% (13 6/12 years)  There was placement of bilateral trans-frontal deep brain  Stimulators, which appear to cross and terminate along the inferior  edge of the globus palladi or several mm below in the ansa  lenticularis.  Low protein diet  Amino acid mixture discontinued  Diffuse hypotonia  Decrease in the extrapyramidal movements  Plasma glycine: 802 µmol/L  Plasma leucine: 55 µmol/L ( normal range: 61-201 µmol/L)  Plasma isoleucine: 31 µmol/L (normal range: 36-108 µmol/L)  Plasma valine: 100 µmol/L (normal range: 134-315 µmol/L)  Serum 2-methylcitrate: 41,947 nmol/L (normal range: 60-228 nmol/L) | **-** Atovaquone 750 mg/5 mL 1200 mg qd via g-tube  - Baclofen 20 mg qPM via g-tube  - Loratadine 5 mg qd via g-tube  - Dextromethorphan 15 mg qd PRN via g-tube  - Clonazepm 0.5 mg BID via g-tube  - Tacrolimus 4.2 mg qp q12 hr  - Cellcept 600 mg BID  - Konsyl 13 gm qd  - Tegretol 100 mg BID  - Melatonin 10 mg qhs PRN  - Benedryl 10 ml qHS PRN |
| 18 and 10/12 years | **Final hospitalization**  *Prior left ventricular ejection fraction of 0.27 (18 years)*  Admitted to the hospital for increased work of breathing  Pulse oximeter oxygen desaturation at home  Fever  Tachypnea  Tachycardia  Heart failure, has been on BiPAP at home  Respiratory distress continued to worsen and he died after approximately 1.5 months of hospitalization  In conjunction with the family, it was decided that he would not be intubated and would not undergo resuscitation; he was given morphine and died shortly thereafter. | *Epi-event medications*  *On admission:*   - Amlodipine (7.5 mg, 2x a day) - Ascorbic acid (500 mg per day) - Aspirin81 (81 mg per day) - Baclofen - Biotin (10 mg per day) - Calcitriol (0.5 microg per day) - Clonazepam (0.5 mg per day) - Cyanocobalamin (1000 microg P.O. per day) - Diazepam (10 mg rectal kit PRN seizure) - Digoxin (125 microg per day) - Furosemide (40 mg, twice a day) - Lactobacillus rhamnosus GG (1 capsule daily) - L-carnitine (1000 mg, 2x a day) - Melatonin (10 mg at bedtime) - Milrinone (0.5 microg per kg per min) - Omega-3 unsaturated fish oil mixture (1200 mg capsule) - Psyllium (6g PRN constipation) - Riboflavin (50 mg a day) - Sertraline (20 mg a day) - Sirolimus (2 mg a day) - Thiamine (100 mg a day) - Ubiquinone (600 mg a day) - Vitamin E (400 IU per day)   *24 h before death:*   - Amlodipine (7.5 mg, 2x a day) - Ascorbic acid (500 mg per day) - Aspirin81 (81 mg per day) - Baclofen - Biotin (10 mg per day) - Calcitriol (0.5 microg per day) - Chlorothiazide (125 mg per day) - Clonazepam (0.5 mg per day) - Cyanocobalamin (1000 microg P.O. per day) - Diazepam (10 mg rectal kit PRN seizure) - Digoxin (125 microg per day) - Furosemide (40 mg, twice a day) - Lactobacillus rhamnosus GG (1 capsule daily) - L-carnitine (1000 mg, 2x a day) - Melatonin (10 mg at bedtime) - Milrinone (0.5 microg per kg per min) - Omega-3 unsaturated fish oil mixture (1200 mg capsule) - Psyllium (6g PRN constipation) - Riboflavin (50 mg a day) - Sertraline (20 mg a day) - Sirolimus (2 mg a day) - Spirinolactone (25 mg a day) - Thiamine (50 mg a day) - Vitamin E (400 IU per day) |
